# A Novel Component Carrier Configuration and Switching Scheme for Real-Time Traffic in a Cognitive-Radio-Based Spectrum Aggregation System

**DOI:** 10.3390/s150923706

**Published:** 2015-09-17

**Authors:** Yunhai Fu, Lin Ma, Yubin Xu

**Affiliations:** 1 Harbin Institute of Technology Communication Research Center, Harbin 150001, China; E-Mails: fucloudsea@163.com (Y.F.); ybxu@hit.edu.cn (Y.X.); 2 Key Laboratory of Police Wireless Digital Communication, Ministry of Public Security, Harbin 150001, China

**Keywords:** RT-CCCS, real-time traffic, spectrum aggregation, resource scheduling, cognitive radio

## Abstract

In spectrum aggregation (SA), two or more component carriers (CCs) of different bandwidths in different bands can be aggregated to support a wider transmission bandwidth. The scheduling delay is the most important design constraint for the broadband wireless trunking (BWT) system, especially in the cognitive radio (CR) condition. The current resource scheduling schemes for spectrum aggregation become questionable and are not suitable for meeting the challenge of the delay requirement. Consequently, the authors propose a novel component carrier configuration and switching scheme for real-time traffic (RT-CCCS) to satisfy the delay requirement in the CR-based SA system. In this work, the authors consider a sensor-network-assisted CR network. The authors first introduce a resource scheduling structure for SA in the CR condition. Then the proposed scheme is analyzed in detail. Finally, simulations are carried out to verify the analysis on the proposed scheme. Simulation results prove that our proposed scheme can satisfy the delay requirement in the CR-based SA system.

## 1. Introduction

Spectrum aggregation (or carrier aggregation) is considered a key enabler for next-generation communication networks, which can meet or even exceed the IMT-Advanced requirement for large transmission bandwidth (40 MHz–100 MHz) and high peak data rate (500 Mbps in the uplink and 1 Gbps in the downlink). In order to meet both the requirements for transmission bandwidth and the utilization of IMT bands, all IMT-Advanced candidate technologies are expected to support spectrum aggregation, within either contiguous or discontinuous spectrum bands [1]. In spectrum aggregation (SA) technology, two or more component carriers (CCs) of different bandwidths in different bands can be aggregated (up to 100 MHz with five CCs of 20 MHz) to support wider transmission bandwidth between the E-UTRAN NodeB (eNB) and the user equipment (UE) [2]. The introduction of SA has required the introduction of new functionalities and modifications to radio resource management (RRM) [3]. With the broadband wireless professional communications stepping into its start-up period, a project called broadband wireless trunking (BWT) was launched in China [4]. SA is one of the key techniques of the BWT. In terms of SA, BWT has some special aspects that are different from those of a mobile cellular system, including lacking enough spectrum bands for broadband data transmission. So, the technique of cognitive radio (CR) is introduced in BWT for SA. CR is considered one of the prominent techniques to improve the spectrum utilization [5] and it is also the key enabling technology that enables next-generation communication networks to utilize the spectrum more efficiently in an opportunistic fashion without interfering with the primary users [6,7,8]. Therefore, CR-based spectrum aggregation is getting more attention.

Amounts of resource scheduling schemes for spectrum aggregation have been proposed in the literature, and these schemes can all be applied to spectrum-aggregation-based communication systems for different performance indicators. Although the aforementioned resource scheduling schemes focus on the different performance indicators to improve the performance of SA, it is worth noting that these performance indicators are not enough to guarantee good system performance. The current resource scheduling schemes for spectrum aggregation have not taken the users’ delay requirement into account. For the BWT system, low transmission latency is the most important performance requirement [4]. Real-time communication and response is one of major technological challenges as we develop the BWT system. Due to this challenge in the development of the BWT system, the current scheduler structures and resource scheduling schemes become questionable and are not suitable for CR-based SA in the BWT system. To satisfy the requirement for low transmission latency of BWT, it is desirable to investigate a new resource scheduling scheme for CR-based SA by taking into account the delay requirement, which motivates this work.

In this paper, we propose a novel resource configuration and switching scheme to meet the challenge of delay requirements in the CR-based SA system, called a component carrier configuration and switching scheme for real-time traffic (RT-CCCS). As a case study, we choose an orthogonal frequency division multiple access (OFDMA)-based wireless communication system for the evaluation of the proposed scheme. Typically SUs are capable of spectrum sensing, but the current spectrum sensing methods cannot guarantee perfect detection of the presence of the primary users (PUs), meaning false alarms and misdetections are unavoidable in real scenarios. False alarms result in less utilization of the spectrum, whereas misdetections result in collisions with the PU transmission. A more appropriate approach for improving the sensing performance of a single user involves outsourcing the spectrum sensing to a low-cost dedicated sensor network [9], which exploits the location diversity of the sensor nodes and improves sensing accuracy and reliability [10]. It is particularly effective in channels experiencing shadowing, fading, and hidden terminal problems. Like cooperative spectrum sensing [11,12,13], the sensor nodes perform spectrum sensing to determine the status of the PUs locally and send their results to the SU, which combines them using a certain rule. This feature of the sensor network is important for resource scheduling in CR-based SA system. So, we assume that the perfect detection of the presence of the PUs can be obtained through the sensor network in this paper. We first introduce a sensor network-assisted resource scheduling structure for SA in the CR condition. Then the proposed scheme is analyzed in detail in terms of performance including the factors that are important to its performance. Finally, simulations are carried out to verify the analysis on the proposed scheme.

The remainder of this paper is organized as follows. Section 2 introduces the related works and Section 3 gives the system model of the proposed scheme. Section 4 designs the proposed scheme in detail. The simulation results are shown and analyzed in Section 5. Finally, some conclusions are drawn in Section 6.

## 2. Related Works

For resource scheduling in SA based systems, there are two different scheduler structures [14,15]: the joint queue scheduler (JQS) and the disjoint queue scheduler (DQS). In the structure of the JQS, each user has only one queue for all CCs, and the CCs share the joint queue of each user. The shared scheduler maps the users’ traffic load to the resource blocks (RBs) on all CCs. Although one user’s data may only be transmitted on some of the CCs, the JQS requires each user to be able to receive a signal from all CCs simultaneously and continuously [15]. It largely increases the signal processing complexity and the power consumption of the UEs [14,16]. Considering that the bandwidth of the SA based system is wide, the JQS implementation leads to high complexity [17]. DQS has a two-step scheduling structure. In the structure of the DQS, each user has one traffic queue on each CC. Through two-layer scheduling, the traffic loads of users are assigned onto certain CCs and the assignment of RBs in each CC is then carried out. When comparing the performance of two-scheduler structures, the performance of the DQS is inferior to that of the JQS in terms of spectral efficiency and resource utilization [15]. JQS is considered the optimal scheduler for the LTE-Advanced system with SA, at the cost of high complexity.

The aggregated spectrum allocation for SA can be decomposed into CC selection and RB assignment phases. CC selection, to assign multiple CCs to users, is the new RRM functionality introduced in LTE-Advanced [15]. As methods for balancing the load across CCs will affect the system performance, the following three most notable CC selection methods to address load balancing have been the main focus of research in the literature: random selection (RS), circular selection (CS), and least load (LL). In RS scheme [18,19], CCs for each UE are chosen randomly from the available CC set by the eNB. From the long-term point of view, it can provide balanced load across CCs. However, at each instant, the load across CCs may not be balanced and the system may suffer from reduced spectrum utilization. The CS scheme [16,17] selects CCs circularly for the traffic data. Compared to RS, CS offers higher throughput and better coverage performance due to better balancing of traffic load over multiple CCs [16]. However, when the users traffic packet sizes are significantly different, the efficiency of CS can be decreased. The LL scheme [14,17,18] allocates the users’ packets to each CC according to the current traffic load of CCs. Since the packets are always allocated to the CC with the lowest traffic load, LL provides better performance than other schemes (e.g., RS and CS) that do not consider the system state information [18]. And there are also other CC selection schemes for the inter-band aggregation. In order to optimize the performance of inter-band SA, the CC selection scheme should not only take the traffic load, but also the radio channel characteristics into consideration as proposed in: inter-band carrier switch method, RSRP based method, and G-factor based method. The inter-band carrier switch method [20] can allocate CCs at lower frequencies *i.e*., 2 GHz first, and then after checking the load on CCs, moves the UEs with the highest channel quality indicator (CQI) in 2 GHz to the 5 GHz band, but this method has increased complexity due to switching and does not consider UEs using CCs in different bands at the same time. The reference signal received power (RSRP) based CC selection scheme [1] allocates the better CC to the UE whose average data rate is relatively small, it is particularly effective for the real-time traffic. The G-factor based carrier selection algorithm [21] makes use of identities of cell-edge UEs based on geometry (G-) factor and is particularly effective in scenarios with large differences in G-factor distributions between CCs. These methods have the good performance for CC selection of SA in the LTE-Advanced system.

Once the users are assigned onto certain CCs by CC selection schemes, the assignment of resource blocks in each CC is carried out. This is the RB assignment phase. For RB assignment schemes, throughput and fairness are considered as target objectives. The Proportional Fair (PF) scheme is selected as benchmark due to its simplicity and good performance [18,22,23]. Since PF is aware of the channel condition for each user, it can exploit the multi-user diversity. In the long term, this scheme can achieve fairness among active users in each CC [23]. But it has no consideration of different SA capability of terminals and does not exhibit good delay performance [24]. In order to improve the fairness between LTE and LTE-Advanced users, Cross-CC PF is proposed in [18,22]. In Cross-CC PF, exchange of information on the past user throughput on each CC is required. Taking the past user throughput over all CCs makes the scheduling metric of LTE-Advanced users smaller compared with that of PF. Cross-CC PF can improve the average user throughputs for LTE users with no degradation in the average cell throughput. Cross-CC PF is the optimal scheduling scheme for a given CC selection [25]. There are also other RB assignment schemes. Round Robin (RR) [14,16,26,27] is the basic scheduling scheme. In order to achieve better fairness among users, a user grouping PF algorithm (UG-PF) is proposed in [28]. With UG-PF, the users in poor channel conditions can obtain throughput improvement and better fairness among users is achieved compared with the PF scheme [29].

However, the aforementioned schemes do not consider that the spectrum aggregation may be carried out in CR networks, and the aforementioned scheduler structures and resource scheduling schemes become questionable and are not suitable for SA in CR networks. It is worth noting that there have been several alternative schemes for spectrum aggregation in the CR-based SA system. In [30], a spatial spectrum sharing-based carrier aggregation (SSS-CA) is considered from a game theory perspective. In SSS-CA, a network operator can not only transmit on its own licensed spectrum but it can also access and aggregate the licensed spectrum of other operators on payment of a certain price. In [31], a CR-based dynamic spectrum access scheme is proposed for an LTE-Advanced supporting carrier aggregation functionality. The scheme is referred to as DSA-CA. DSA-CA can activate and deactivate dynamically assigned CCs at an eNB depending on the traffic load of its cells. A spectrum aggregation algorithm and two spectrum allocation algorithms are proposed in [32]; the proposed spectrum aggregation algorithm can significantly increase the available bandwidth that secondary users (SUs) can access, and the proposed spectrum allocation algorithms can markedly improve the spectrum utilization efficiency. The authors in [33] have proposed a SAGCA algorithm based on the capacity of CR equipment. SAGCA can satisfy users’ bandwidth requirements and utilize the available spectrum more efficiently. In [34], a spectrum aggregation algorithm called CCASA is proposed, which takes spectrum aggregation and channel state information into consideration. CCASA enables SUs to achieve a higher transmission rate and greater total throughput. A spectrum allocation algorithm aware spectrum aggregation is proposed in [35], and the spectrum utilization is improved by aggregating different idle spectrum fragments and assigning them to SUs opportunistically. In [36], a positional proportional fairness (PPF) model based on spectrum aggregation is proposed; its purpose is to solve the problem of unfairness resources aggregation caused by disadvantage positions, and the fairness and bandwidth jitter of PPF are better than the traditional proportional fairness in shadow. The authors in [37] have proposed a spectrum-aggregation-based cooperative routing protocol (SACRP) for CR Ad-Hoc Networks. SACRP considers two classes of cooperative routing protocols: *Class A* for power minimization or throughput maximization, and *Class B* for reducing the end-to-end delay. *Class A* aggregates multiple channels and selects suitable relay nodes, and therefore achieves higher power efficiency and throughput. *Class B* reduces the number of re-transmissions by selecting the relay nodes with better channel conditions, and therefore reduces the end-to-end delay. However, these schemes have the common limitation that they can not guarantee performance in terms of the users’ delay for SA in CR networks.

## 3. System Model

In this paper, the OFDMA-based CR system is considered. Each CR user of the system is able to receive data from multiple CCs, sense the PUs signal of the CCs, estimate the channel quality on all CCs and feed back the CQI to the transmitter. For each CC, its time and frequency resource is divided into many RBs.

### 3.1. Structure of JQS and DQS

**Figure 1:**
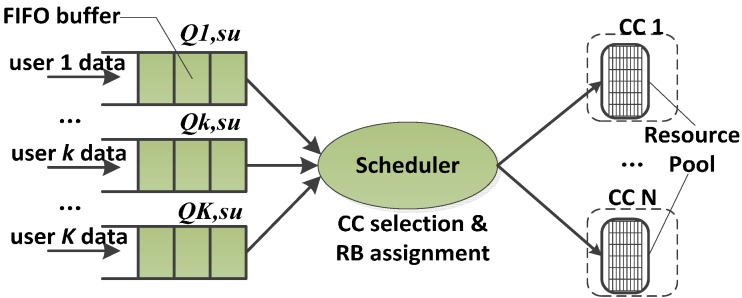
Structure of JQS.

JQS, as shown in Figure 1, is one of the straightforward scheduler structures to manage the multiple CCs. Each user is assigned a separate buffer to store the user data and the buffer is organized in a first-in first-out (FIFO) manner. We denote the buffer of the user *k* with *Q_k,su_*. The resource pool is composed of RBs which belong to the CCs. JQS combines the multiple carriers together as one carrier to allocate RBs. This is the same as the scheduling in the traditional single-carrier systems. Considering that the bandwidth of the SA system is wide, the JQS-based implementation can lead to increased complexity. Therefore, the weakness of the JQS is its high complexity. However, the JQS maximizes the spectral efficiency and achieves the saturated resource utilization. In terms of performance, the JQS is the optimal scheduler structure for SA.

**Figure 2:**
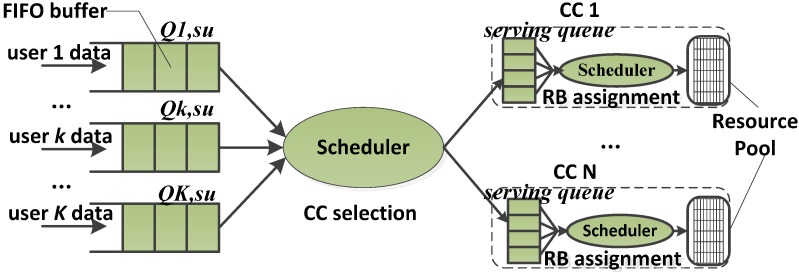
Structure of DQS.

The structure of the DQS is shown in Figure 2. In each CC, there is a serving queue for each user. The resource pool is composed of RBs which belong to the CC that are controlled by RB assignment scheduler. In the DQS, each user has one traffic queue on each CC. Obviously, the DQS needs two level scheduling. The first level is CC selection, which is in charge of the user allocation between the CCs. The second level is RB assignment, which carries out the assignment of RBs in each CC. DQS is the simplest scheduler structure for the SA systems, but it cannot fully utilize the resource in the SA systems and is inefficient especially when the packets are large and sparse.

The performance of the DQS is found to be inferior than that of the JQS in terms of spectral efficiency and resource utilization. The average burst rate supported in the DQS is always lower than that of the JQS, and the gain in average data rate of the JQS over the DQS decreases with the number of users per sector due to a decrease in difference in frequency selective gains.

### 3.2. Structure of Proposed Scheme

**Figure 3:**
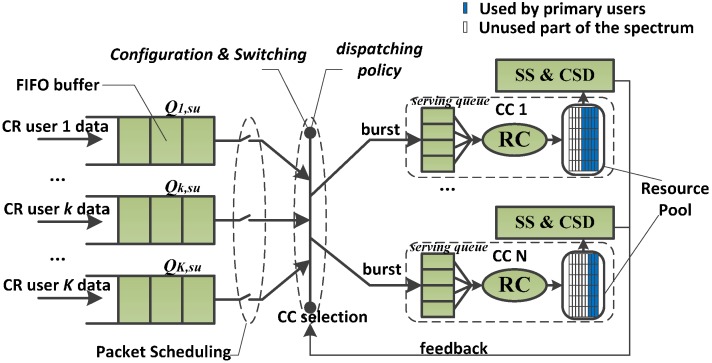
The resource scheduling structure of the RT-CCCS scheme.

In CR, PUs can influence greatly the performance of CC selection schemes. This means that sensing the PUs’ signal is taken into account when we design the resource scheduling scheme. The proposed framework of the resource scheduling is illustrated in Figure 3 based on the structure SBLS proposed in [12]. The functionality of the resource controller (RC) is to manage the users’ buffers. In each RC, there is a serving queue for each user. The resource pool of RC is composed of RBs which belong to the CC that are controlled by RC. Each RC reads data from its serving queue to form transmission blocks for its serving users. The role of spectrum sensing and channel state detection (SS & CSD) is to sense PUs, estimate the channel quality of CCs and feed back the result to the CC selection. The sensor network is adopted in SS & CSD for sensing the presence of PUs; the sensor nodes perform sensing to determine the status of the PUs locally and send their results to the SU, which use the SA technology for SU data transmission in the CR network. The results of SS & CSD are the presence of PUs and the channel quality of CCs, and these results are fed back to the CC selection.

We take each CC as an independent carrier, so there should be the same number of RCs and CCs in the system. The resource that is contained in each RC’s resource pool is from only one of the CCs. The whole resource scheduling structure is a three-level scheduling structure. Unlike the structure of the DQS, the first level of the resource scheduling structure is the packet scheduling for SUs’ data, which carries out the SUs’ data packet and sends the packet to the second level; the size of the packet is determined by the result of the second level. The second level is the CC selection, which is in charge of the CR user allocation between the CCs. There is a component carrier configuration and switching process and a dispatching process in the CC selection. The task of the component carrier configuration and switching process is to configure and switch the number of CCs based on the SUs’ traffic QoS guarantees. The third level is in charge of the burst data transmission, and is handled by each RC. In the proposed resource scheduling structure, CR users are not served by the fixed RC any more. The dominant RC of one CR user can be changed in the burst-level but its number is still one. This means that, among all the serving queues for CR user *k* in different RCs, there is only one filled with one burst for CR user *k*. The size of the burst is calculated based on the results of SS & CSD.

The specific resource scheduling procedure in the proposed resource scheduling structure is as follows. At the beginning of the procedure, the CR users’ buffers are firstly updated if there are new arrival SU data and the packet scheduling is carried out. After that, the dispatching condition is checked. Before the dispatching, the component carriers configure and switching process is implemented to configure and switch the number of CCs based on the SUs’ traffic QoS guarantees. The dispatching will happen only if some CCs are not used by PUs and there are still CR users’ data waiting for transmission. RT-CCCS selects one of the idle CCs as the dominant CC and assigns the user data with priority to the dominant CC. Every time only one of the idle CCs will be selected as the dominant CC, only the burst in the head of CR user buffer will be dispatched, and the burst will be dispatched to the dominant CC. Repeat this cycle until all CCs are not idle. When the data transmission in a CC has not been finished or there is a PU signal in the CC, the corresponding CC cannot be selected as the dominant CC.

In essence, the way for the proposed resource scheduling structure to improve the resource utilization is to decrease the traffic dispatching granularity and change the dominant RC when the PUs’ signal arises in the CCs. This means the traffic load across the CCs can be better balanced in a CR-based SA system. It is impossible for the proposed resource scheduling structure to outperform the JQS, but its complexity is lower than that of the JQS. Greater resource utilization of the proposed resource scheduling structure over the DQS is imaginable. Although it increases the complexity of the proposed resource scheduling structure, this complexity increase is moderate. The resource scheduling structure of the RT-CCCS scheme also has some advantages over SBLS. In SBLS, there are the same number of resource schedulers (RSs) and CCs, and each RS in SBLS carries out RB assignment and the user’s data transmission control. In the resource scheduling structure of RT-CCCS, there are also the same number of RCs and CCs, but each RC only carries out the user’s data transmission control. The function of RB assignment is implemented by the packet scheduling in the resource scheduling structure of RT-CCCS, and there is only one packet scheduling module in the structure of RT-CCCS. Hence the proposed resource scheduling structure of RT-CCCS shows lower complexity than SBLS. In other hand, when the user data is assigned to a certain CC during the burst transmission, the user data will wait for data transmission in SBLS if the certain CC is suddenly occupied by PUs in CR networks, but the user data will be switched to another idle CC in the structure of RT-CCCS instead of waiting for data transmission. Thus, the proposed structure of RT-CCCS has better delay performance than SBLS in CR networks.

## 4. Design of RT-CCCS

**Figure 4:**
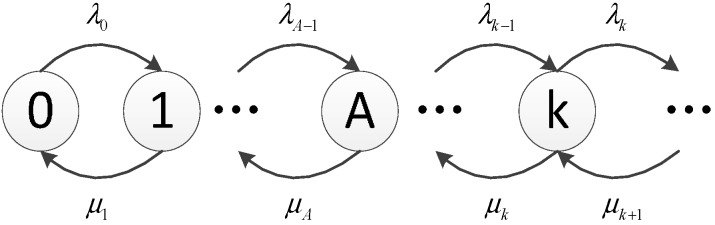
The birth-death process in queueing modeling.

The traffic model plays an important role in analyzing the performance of resource scheduling schemes. The arrival, or departure, of users in a network is usually modeled as a birth-death process, as shown in Figure 4. *λ_k_* is the arrival rate going from state *k* to state *k +* 1 (a ‘birth’), *μ_k_* is the departure rate of going back to state *k −* 1 (a ‘death’). The birth-death process is a special case of a continuous-time Markov process, where the states represent the current number of active users and transitions are between neighboring states. The ‘birth’ is the transition towards increasing the number of active users by 1, and the “death” is the transition towards decreasing the number of active users by 1. This is a typical M/M/1/A process, where the arrival of users follows the Poisson distribution, and the service time follows a negative exponential distribution. *A* is the corresponding maximum number of users in the system. Although the negative exponential distribution of service time is usually assumed for voice calls, it can also roughly represent the time for SUs to transmit data in CR-based SA. The latter is the case considered here, and the assumptions are later verified via simulation results, showing a good match.

In this section, the proposed resource scheduling structure is described and analyzed in detail. In this paper, we will use the following notation:

$\lambda_{i,pu}$ Arrival rate of PUs on the *i*th CC, in users per second
$\mu_{i,pu}$ Service rate of PUs on the *i*th CC, in users per second
$\lambda_{su}$ Arrival rate of SUs, in users per second
$\mu_{su}$ Service rate of SUs, in users per second
$C_{i}$ The throughput of the *i*th CC in Mbps, assuming the CC is fully loaded
F Payload size for each SU packet transmission, in Mbits
$L_{i,su}$ The load of SUs which has been transmitted on the *i*th CC
E(•) Mathematical expectation operation
D(•) Variance operation

### 4.1. Packet Scheduling

The first level of the proposed resource scheduling structure is packet scheduling for SUs’ data. In packet scheduling study, we select the Cross-CC PF, following the description as outlined in [13]. The Cross-CC PF takes the statistics from all CCs into consideration, so it can achieve an overall better resource allocation. In this paper, the assignment of the user *k* should meet the following scheduling metric:

(1) $$k^{*}=\arg\underset{k}{\max}\left(M_{k}\right)$$

where k^*^ is the selected user, and *M_k_* is the priority metric. The priority metric is calculated by dividing the instantaneous transmission data rate of the current dominant CC by its past throughput over all aggregated CCs,

(2) $$M_{k}=\frac{R_{dominant}}{{\tilde{R}}_{k}}$$

where *R_dominant_* is the instantaneous transmission data rate of the current dominant CC, *N* is the number of CCs which is used for SA, and ${\tilde{R}}_{k}$ is the average delivered throughput for user *k* in the past. The difference from Cross-CC PF proposed in [13] is that the packet scheduling is carried out before CC selection.

### 4.2. CC Selection

In this paper, we consider a standard M/M/1 process for PUs, where the arrival of PUs in the CR-based SA system follows the Poisson distribution, and the service time of PUs follows a negative exponential distribution. Before the dispatching, the component carriers configure and switching process is implemented to configure and switch the number of CCs based on the SUs’ traffic QoS guarantees. Then, the dispatching process is carried out.

#### 4.2.1. Component Carriers Configuration and Switching Process

The traffic model plays an important role in analyzing the performance of resource scheduling schemes in SA systems. We therefore consider a M/M/S/A process in this paper, which is a dynamic traffic model with Poisson arrival and used in [10]. In this process, the arrival of SUs follows the Poisson distribution, and the service time follows a negative exponential distribution (M for Markov). S is the number of active SUs, and A is the corresponding maximum number of SUs in the CR-based SA system. In SA, all CCs are regarded as one channel for users, so S is equal to 1 in this paper.

The admission control in CR-based SA system is assumed to limit the SUs’ number of CCs to maximum *A* SUs. This leads to the following arrival rate:

(3) $$\lambda_{su}=\{\begin{array}{lll} \lambda & & 0\leq k<A\\ 0 & & k\geq A \end{array}$$

The service rate, which is the average number of SUs who finish their transmissions during one second, can then be expressed as

(4) $$\mu_{su}=\{\begin{array}{lll} \frac{C_{eq}}{F} & & 0\leq k<A\\ 0 & & k\geq A \end{array}$$

Because the SUs and PUs coexist in each CC, the SUs’ data transmission time is affected by PUs. *T_i_* denotes the SUs’ data transmission time of the *i*th CC, *L_i,su_* denotes the length of the SUs’ data transmitted in the *i*th CC, *T_i,pu_* denotes the PUs’ data transmission time of the *i*th CC, and *C_i_* is the data throughput of the *i*th CC, so the each SU’s data transmission time of the *i*th CC can be expressed as

(5) $$T_{i}=\frac{L_{i,su}}{C_{i}}+T_{i,pu}$$

Based on the principle of dispatching policy, the data transmission time of each SUs’ packet in every CCs is also equal. *T* denotes the SUs’ data transmission time when SUs aggregate the all CCs for data transmission, thus

(6) $$T=T_{i},i=1,2,\ldots ,N$$

Based on Equations (5) and (6), we can obtain the following expression:

(7) $$\left\{ \begin{array}{l} L_{i,su}=TC_{i}-T_{i,pu}C_{i}\\ \sum_{i=1}^{N}L_{i,su}=F \end{array}\right.$$

Thus, the SUs’ data transmission time *T* can be obtained when SUs use the CR-based spectrum aggregation for data transmission.

(8) $$T=\frac{F}{C}+\sum_{i=1}^{N}\frac{C_{i}T_{i,pu}}{C}$$

where

(9) $$C=\sum_{i=1}^{N}C_{i}$$

We can find that *T* is related to the payload size *F*, data throughput *C_i_* of each CC, and PUs’ data transmission time *T_i,pu_* of each CC. *T_i,pu_* obeys Erlang distribution and its order is related to *T*. Thus, the expectations of *T_i,pu_* and $T_{i,pu}^{2}$ can be obtained.

(10) $$\left\{ \begin{array}{l} \text{E}(T_{i,pu})=\frac{\lambda_{i,pu}}{\mu_{i,pu}}\text{E}\left(T\right)\\ \text{E}(T_{i,pu}^{2})=\frac{2\lambda_{i,pu}}{\mu_{i,pu}^{2}}\text{E}\left(T\right)+\frac{\lambda_{i,pu}^{2}}{\mu_{i,pu}^{2}}\text{E}\left(T^{2}\right) \end{array}\right.$$

So, based on Equations (8) and (10), we can calculate the expectation and variance of the SUs’ data transmission time *T*, as follows.

(11) $$\text{E}(T)=\frac{F}{C-\sum_{i=1}^{N}\frac{C_{i}\lambda_{i,pu}}{\mu_{i,pu}}}$$

(12) $$D(T)=\frac{F\sum_{i=1}^{N}\frac{2C_{i}^{2}\lambda_{i,pu}}{C^{2}\mu_{i,pu}^{2}}}{C(1-\sum_{i=1}^{N}\frac{C_{i}\lambda_{i,pu}}{C\mu_{i,pu}})(1-\sum_{i=1}^{N}\frac{C_{i}^{2}\lambda_{i,pu}^{2}}{C^{2}\mu_{i,pu}^{2}})}$$

A M/G/1 process is considered for SUs in the CR-based SA, the arrival of SUs follows the Poisson distribution, and the service times are independent random variables have a common probability distribution. The service times are independent of the arrival process. *T_w_* denotes the average delay of the SUs’ data transmission time. Based on the Pollaczek–Khintchine formula, we can obtain the expression of *T_w_* as follows.

(13) $$T_{w}=\text{E}(T)+\frac{\lambda (D(T)+{\text{E}}^{2}(T))}{2(1-\rho_{su})}$$

where

(14) $$\rho_{su}=\lambda \text{E}(T)$$

If the expectation and variance of the SUs’ data transmission time *T* is known, we can get the average delay of the SUs’ data transmission time when SUs transmit data by SA.

#### 4.2.2. Dispatching Process

The probability of no primary users on the *i*th CC can be expressed as follows:

(15) $$P_{0,i}=1-\frac{\lambda_{i,pu}}{\mu_{i,pu}}$$

where $P_{0,i}$ is the probability of no primary users on the *i*th CC. The probability can be obtained based on the arrival rate and service rate of PUs on the *i*th CC. SUs can only use the idle part of the PUs’ spectrum for data transmission, so we define an equivalent throughput *C_i,eq_* for SUs on the *i*th CC:

(16) $$C_{i,eq}=P_{0,i}C_{i}$$

From the long term point of view, this means that the *i*th CC can provide a throughput *C_i,eq_* for SUs. If there are *N* CCs, the throughput of CCs can be obtained:

(17) $$C_{eq}=\sum_{N}C_{i,eq}$$

where *C_eq_* is the equivalent throughput of CCs, and *N* is the number of CCs which is used for SA.

We allocate the SUs’ packets to each CC according to the current channel state and SUs’ traffic load of CCs. The principle of the dispatching policy is that the SUs’ data transmission time of each CC is equal, as the following expression shows.

(18) $$\left\{ \begin{array}{l} \frac{L_{i,su}}{C_{i,eq}}=\frac{L_{i+1,su}}{C_{i+1,eq}},i=1,...,N-1\\ \sum_{N}L_{i,su}=KF \end{array}\right.$$

where *K* is the SUs’ number, *F* is fixed payload size for each SU’s packet transmission, and *L_i,su_* is the load of SUs which has been transmitted on the *i*th CC. So the SUs’ load of the *i*th CC is

(19) $$L_{i,su}=\frac{C_{i,eq}}{C_{eq}}KF$$

So, the selection of the dominant CC is performed as

(20) $$i^{*}=\arg\underset{i}{\min}\left(\frac{L_{i,su}}{C_{i,eq}}\right)$$

This means that the CC that has the least data transmission time for SUs will be selected. The dispatching policy assigns the different SUs’ load to each CC according to the channel state in the presence of PUs, it improves the spectrum resource utilization, and the SUs’ traffic load across the CCs can be better balanced in CR-based SA system.

### 4.3. Burst Data Transmission

The third level is in charge of the burst data transmission, which is handled by each RC. In the proposed resource scheduling structure, CR users are not served by the fixed RC anymore. The dominant RC of one CR user can be changed in the burst level but its number is still one. This means that, among all the serving queues for CR user *k* in different RCs, there is only one of them filled with one burst of CR user *k*. The size of the burst is calculated based on the results of SS & CSD.

## 5. Implementation and Performance Analysis

In this section, simulations are carried out to evaluate the performance of our proposed scheme. We consider a static OFDMA based system for SA, and the wireless channel is modeled including distance-dependent path loss, shadowing fading and multipath Rayleigh fading. There are two component carrier configuration and switching schemes used with the comparative. One is the proposed RT-CCCS scheme, and the other is an equivalent-throughput-based component carrier configuration and switching (Ceq-CCCS) scheme, whose principle can be expressed as follows:

(21) $$C_{eq}\geq \frac{F}{T_{delay}}$$

where *T_delay_* is the delay requirement which is required for SUs’ data transmission. The two schemes have the same scheduler structure which is proposed in Section 2. The simulation parameters are summarized in Table 1.

**Table 1:** Simulation settings.

Parameter	Setting/Description
Carrier aggregation pattern	3 MHz per CC
Number of RBs per CC	50 (12 subcarriers per RB)
Sub-frame duration	1 ms (11 OFDM data symbols plus 3 control symbols)
Modulation and coding schemes	64QAM (5/6)
Traffic model	M/M/1 for PUs
Delay requirement	200 ms
Admission control constraint	Maximum 50 SUs for the CCs

To facilitate performance analysis, the PUs’ arrival rate and service rate in each CC are same, *λ_pu_* denotes the PUs’ arrival rate and it changes from 0 to 20 users per second, and the PUs’ service rate is equal to 50 users per second in each CC. A long simulation run (with duration of 100 seconds) is needed.

The following measures are used in our study as performance indicators:

**Average delay time**: the average period from SUs’ arrival to complete departure.

**Number of CCs used**: the number of CCs which are used for SU data transmission in the SA based system.

**Resource efficiency**: the rate between the resource used for SU data transmission and the resources of all CCs which can be used for SUs’ data transmission.

### 5.1. Number of CCs Is Not Fixed

The number of CCs is not fixed, which means that SUs can configure the different number of CCs for SA and the SUs’ data transmission can be switched between CCs, depending on the SUs’ delay requirement. Assume there are 10 CCs and each CC has same condition as shown in Table 1. *F* is fixed and equal to 1 Mbits.

**Figure 5:**
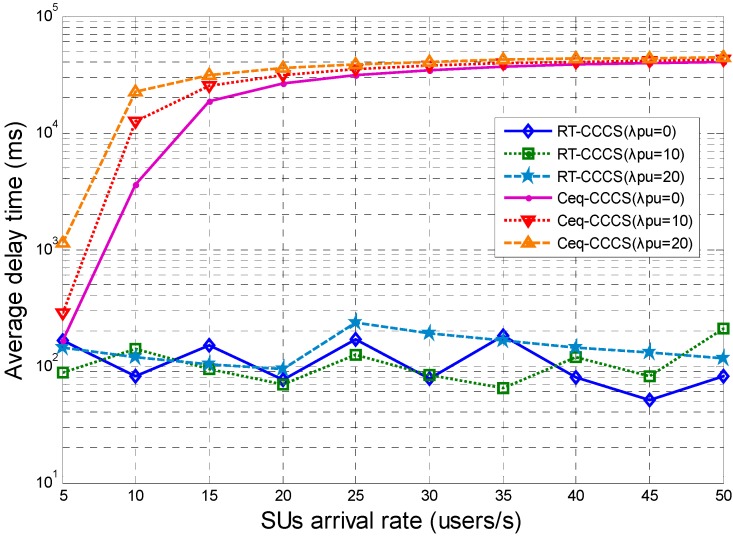
Average delay time of RT-CCCS and Ceq-CCCS under different SU and PU arrival rates (*F* is fixed and equal to 1 Mbits).

The simulation results presented in Figure 5 show the average delay time of the proposed RT-CCCS scheme and the Ceq-CCCS scheme with different SU and PU arrival rates. From Figure 5, it can be observed that the average delay time of Ceq-CCCS is considerably higher than that of RT-CCCS when the SUs’ arrival rate is higher than 5 users per second. As the SUs’ arrival rate increases, the average delay time of RT-CCCS is roughly stable overall and almost less than 200 ms, but the average delay time of Ceq-CCCS is almost up to 40,000 ms when the SUs’ arrival rate changes from 5 users per second to 50 users per second. This means that RT-CCCS has the better delay performance than Ceq-CCCS in the CR-based SA, and RT-CCCS can satisfy the request of the delay and is more suitable for CC configuration and switching in the CR-based SA system. These are the results that are expected in this paper.

**Figure 6:**
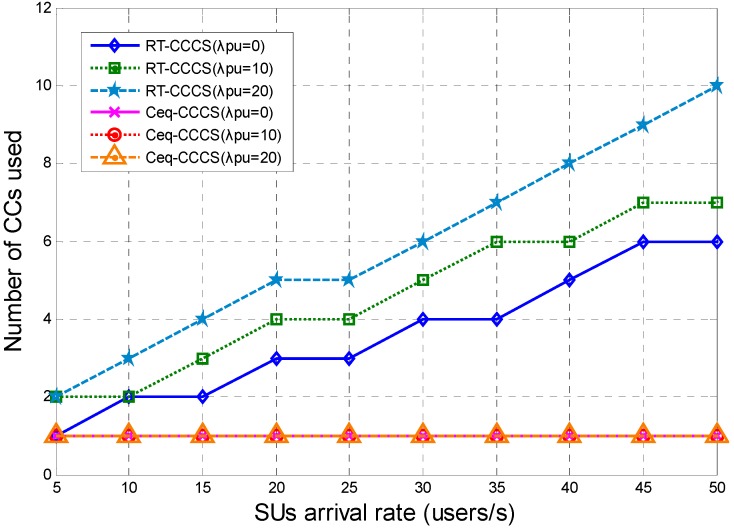
Number of CCs used for RT-CCCS and Ceq-CCCS under different SU and PU arrival rates (*F* is fixed and equal to 1Mbits).

The numbers of CCs used for RT-CCCS and Ceq-CCCS under different SU and PU arrival rates are shown in Figure 6. From Figure 6, we can see that the number of CCs used for RT-CCCS varies with the SUs arrival rate and PUs arrival rate, but that of Ceq-CCCS does not. The number of CCs used for Ceq-CCCS may vary with the PU arrival rate, but the PU arrival rate or payload size *F* is not large enough and the number of CCs used for Ceq-CCCS does not vary with the PUs’ arrival rate. This means that RT-CCCS can configure the different CCs and switch the SUs’ data transmission between the CCs based on the PUs’ arrival rate of each CC and SU arrival rate. The delay performance of RT-CCCS is better than that of Ceq-CCCS at the cost of more resources.

**Figure 7:**
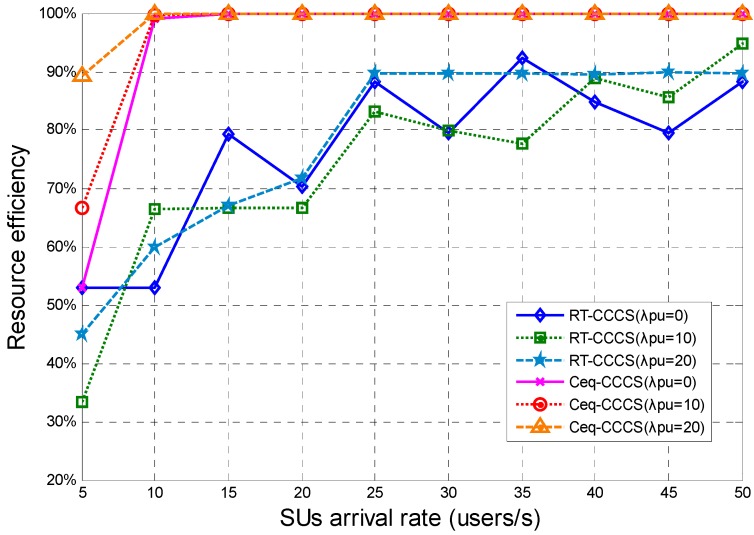
Resource efficiency of RT-CCCS and Ceq-CCCS under different SU and PU arrival rate (*F* is fixed and equal to 1 Mbits).

Figure 7 presents the resource efficiency of the RT-CCCS scheme and the Ceq-CCCS scheme under different SU and PU arrival rates. The simulation results show that the resource efficiency of RT-CCCS is significantly lower than that of Ceq-CCCS. The resource efficiency of Ceq-CCCS is increased with increasing SU arrival rate, and it is up to 100% when the SU arrival rate is larger than 10 users per second. The resource efficiency of RT-CCCS is also increased with increasing SU arrival rate, and it is about 90% when the SU arrival rate is larger than 25 users per second. The delay performance of RT-CCCS is better than that of Ceq-CCCS at the cost of low resource efficiency.

Therefore, we can draw the conclusion that the RT-CCCS scheme can satisfy the request of the delay and is suitable for CC configuration and switching in the CR-based SA system. The delay performance of RT-CCCS is better than that of Ceq-CCCS at the cost of more resources and low resource efficiency.

### 5.2. Number of CCs Is Fixed

The number of CCs is fixed, which means that SUs cannot meet the SUs’ delay requirement by aggregating the different number of CCs. SUs can adjust the payload size *F* for each SU’s packet transmission. Assume there are 4 CCs and all of CCs are used for the SUs’ data transmission. Each CC has same condition as shown in Table 1. Here, we only analyze the performance of the proposed RT-CCCS scheme when the number of CCs is fixed and equal to 4.

**Figure 8:**
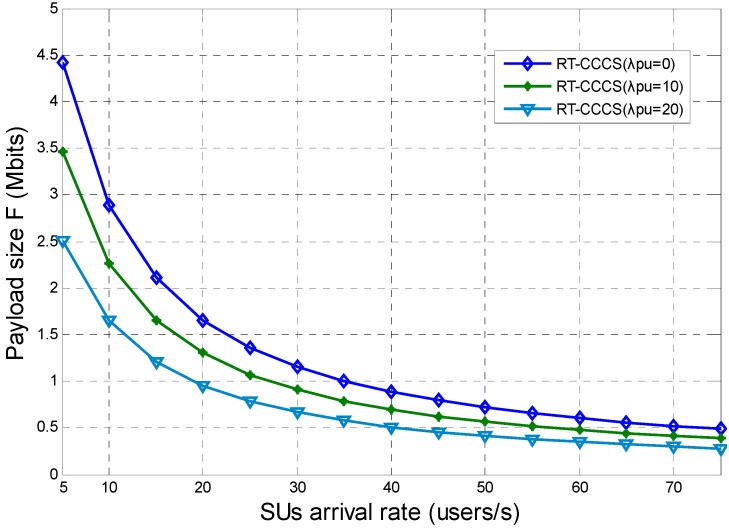
Maximum payload size of each SU packet which can be reached within the system delay requirement for RT-CCCS under different SU and PU arrival rates.

The simulation results presented in Figure 8 show the maximum payload size of each SU packet which can be reached within the system delay requirement for RT-CCCS under different SU and PU arrival rates. In this simulation, the system delay requirement is 200 ms. From Figure 8, it can be observed that the maximum payload size of each SU’s packet is related to the SU and PU arrival rates. The maximum payload size decreases with the increase of the SU or PU arrival rate. This means that the maximum payload size of each SU’s packet should be reduced to meet the system delay requirement when the SU or PU arrival rate increases.

**Figure 9:**
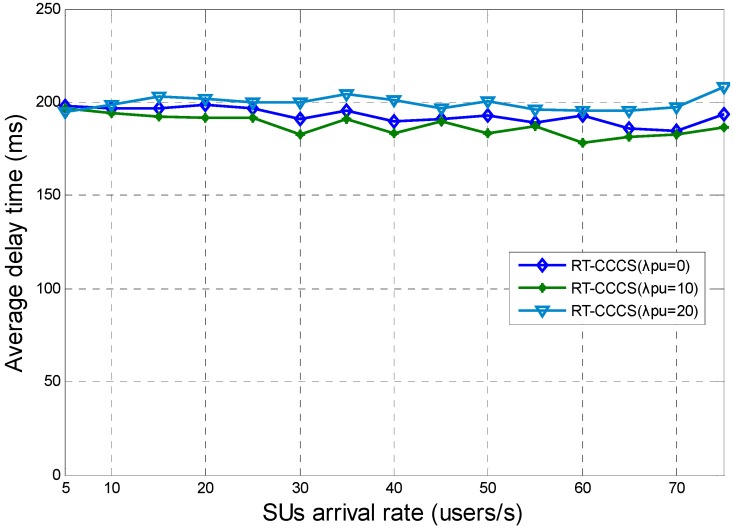
Average delay time of RT-CCCS under different SU and PU arrival rates when *F* is equal to the corresponding maximum payload size of each SU’s packet.

When *F* is equal to the corresponding maximum payload size of each SU’s packet, the average delay times of RT-CCCS under different SU and PU arrival rates are shown in Figure 9. The corresponding maximum payload size of each SU packet is each SU packet’s maximum payload size which can be reached within the system delay requirement under different SU and PUs arrival rates, and it is shown in Figure 8. In this simulation, the system delay requirement is 200 ms. The traffic dispatching granularity of RT-CCCS is a sub-frame duration and it is 1 ms. PUs will occupy the whole sub-frame duration when PUs’ signals appear in the middle of a sub-frame duration. This indicates that the actual corresponding maximum payload size is lower than that calculated by Equations (11)–(13). Therefore, when there are PUs in the CR-based SA system, we adjust the actual corresponding maximum payload size by subtracting a fixed size from the maximum payload size shown in Figure 8. This leads to an average delay time of RT-CCCS (*λ_pu_* = 0) between other two cases, and this result is acceptable. We can see that the average delay time of RT-CCCS is approximately equal to 200 ms under different SU or PU arrival rates. These are the results that are expected in this paper.

**Figure 10:**
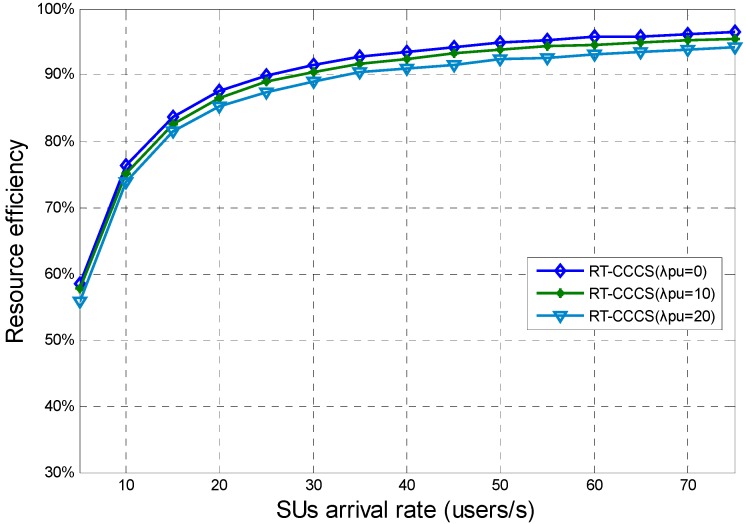
Resource efficiency of RT-CCCS under different SU and PU arrival rates when *F* is equal to the corresponding maximum payload size of each SU’s packet.

Figure 10 presents the resource efficiency of RT-CCCS under different SU and PU arrival rates when *F* is equal to the corresponding maximum payload size of each SU’s packet. From Figure 10, we note that the SU and PU arrival rates have different effects on the resource efficiency of RT-CCCS. The different effects can be clearer and more obvious than that in Figure 7. The simulation results show that the resource efficiency of RT-CSSS is more influenced by the SU arrival rate than the PU arrival rate. The resource efficiency of RT-CSSS increases with the SU arrival rate, and it is much different with the PU arrival rate.

When the payload size of each SU packet is less than the corresponding maximum payload size, the average delay time of RT-CCCS can meet the delay requirement. Therefore, we can draw the conclusion that the proposed RT-CCCS scheme can be used to meet the SUs’ delay requirement when SUs adopt the SA technology in the CR network.

## 6. Conclusions

In this paper, a novel component carrier configuration and switching scheme, RT-CCCS, is proposed and analyzed. We introduce a scheduler structure for resource scheduling in a CR-based SA system; the scheduler structure is a three-level scheduling structure and the RT-CCCS scheme is adopted in the second level of the scheduler structure. When the PU arrival rate and service rate in each CC are obtained, we can use the RT-CCCS scheme to aggregate the appropriate CCs for the corresponding delay requirement based on the SU arrival rate and payload size of each SU packet. Simulation results prove that our proposed scheme can satisfy the SU delay requirement and better performance can be obtained over the traditional methods at the cost of more resources and low resource efficiency in the CR-based SA system.

Although we regard sacrificing resource efficiency as the cost in the RT-CCCS scheme, it will not be a problem when we serve for real-time service first and then make non-real-time service share the residual capacity of the CR-based SA system. With the precondition of the delay requirement for SUs’ real-time service, the transmission of SUs’ non-real-time service can increase the resource efficiency of the CR-based SA system.
